# Preparation, characterization and *in vitro* efficacy of magnetic nanoliposomes containing the artemisinin and transferrin

**DOI:** 10.1186/2008-2231-22-44

**Published:** 2014-05-28

**Authors:** Amir Gharib, Zohreh Faezizadeh, Seyed Ali Reza Mesbah-Namin, Ramin Saravani

**Affiliations:** 1 Department of Laboratory Sciences, Borujerd Branch, Islamic Azad University, Borujerd, Iran; 2 Department of Clinical Biochemistry, Faculty of Medical Sciences, Tarbiat Modares University, Tehran, Iran; 3 Department of Biochemistry, School of Medicine, Zahedan University of Medical Sciences, Zahedan, Iran

**Keywords:** Artemisinin, Transferrin, Liposome, MCF-7 cells, *In vitro*

## Abstract

**Background:**

Artemisinin is the major sesquiterpene lactones in sweet wormwood (*Artemisia annua* L.), and its combination with transferrin exhibits versatile anti-cancer activities. Their non-selective targeting for cancer cells, however, limits their application. The aim of this study was to prepare the artemisinin and transferrin-loaded magnetic nanoliposomes in thermosensitive and non-thermosensitive forms and evaluate their antiproliferative activity against MCF-7 and MDA-MB-231 cells for better tumor-targeted therapy.

**Methods:**

Artemisinin and transferrin-loaded magnetic nanoliposomes was prepared by extrusion method using various concentrations of lipids. These formulations were characterized for particle size, zeta potential, polydispersity index and shape morphology. The artemisinin and transferrin-loading efficiencies were determined using HPLC. The content of magnetic iron oxide in the nanoliposomes was analysed by spectrophotometry. The in vitro release of artemisinin, transferrin and magnetic iron oxide from vesicles was assessed by keeping of the nanoliposomes at 37°C for 12 h. The in vitro cytotoxicity of prepared nanoliposomes was investigated against MCF-7 and MDA-MB-231 cells using MTT assay.

**Results:**

The entrapment efficiencies of artemisinin, transferrin and magnetic iron oxide in the non-thermosensitive nanoliposomes were 89.11% ± 0.23, 85.09% ± 0.31 and 78.10% ± 0.24, respectively. Moreover, the thermosensitive formulation showed a suitable condition for thermal drug release at 42°C and exhibited high antiproliferative activity against MCF-7 and MDA-MB-231 cells in the presence of a magnetic field.

**Conclusions:**

Our results showed that the thermosensitive artemisinin and transferrin-loaded magnetic nanoliposomes would be an effective choice for tumor-targeted therapy, due to its suitable stability and high effectiveness.

## Background

Artemisinin is the major sesquiterpene lactones in sweet wormwood (*Artemisia annua* L.), and it possesses a range of medicinal properties including anti-malaria and anti-cancer activities [1]. This compound has an endoperoxide bridge in its structure. When in contact with high iron concentrations, the molecule releases reactive oxygen species [2]. It has been documented that cancer cells need high iron levels to proliferate; hence cancer cells typically absorb a significantly larger amount of transferrin than normal cells and were more susceptible to artemisinin cytotoxicity [3]. However, the insolubility of artemisinin in the water and its non-selective targeting towards cancer cells could limit its use [4]. Later studies demonstrated that the encapsulation of artemisinin in drug delivery systems and covalently tagging artemisinin to transferrin could partly resolve the aforementioned problems, but it could not resolve the specific targeting of tumors [2,5]. In this case, other options such as preparing of magnetic nanoliposomes containing artemisinin and transferrin can be considered.

Magnetic liposomes are spherical and colloidal vesicles entrapping magnetic iron oxide (Fe_3_O_4_) and may range from tens of nanometers to several micrometers in diameter [6]. Magnetic liposomes loaded with magnetic iron oxide were used as an important drug delivery system, because they can transport drugs to the therapeutic site for cancer treatment [7]. These carriers could congregate around the magnetic site and act as "intelligent" drug delivery systems [8]. Moreover, magnetic liposomes have multi-functionality applications such as image contrasting in magnetic resonance imaging (MRI) and hyperthermia cancer therapy [9].

We speculate that encapsulation of artemisinin and transferrin in magnetic nanoliposomes could increase the artemisinin and transferrin stability, whilst improving selective targeting towards cancerous tumors trough magnetic attraction and thermosensivity. The ability of co-encapsulation of artemisinin and transferrin in magnetic nanoliposomes has not yet been studied. The primary objective of this study was to prepare the thermosensitive and non-thermosensitive magnetic nanoliposomes containing artemisinin and transferrin and evaluate their physicochemical properties. A secondary objective was to investigate cytotoxicity of prepared nanoliposomes against MCF-7 and MDA-MB-231 cells using MTT assay.

## Methods

### Chemicals

Artemisinin (purity ≥ 98%), soy phosphatidylcholine (SPC), distearoyl phosphatidylcholine (DSPC), dipalmitoyl phosphatidylcholine (DPPC), cholesterol (CHOL), magnetic iron oxide, tamoxifen (an anti-cancer drug, as the positive control, purity ≥ 99%) and human transferrin (partially iron saturated, purity ≥ 98%) were obtained from Sigma (USA). Acetonitrile and ammonium sulphate were purchased from Merck (Germany). Alpha-modified Eagle's medium (aMEM) and fetal bovine serum (FBS) were obtained from Gibco (USA).

### Cell culture

MCF-7 (NCBI C135) and MDA-MB-231 (ATCC HTB-26) breast cancer cell lines were purchased from National Cell Bank of Iran (Pasteur Institute, Tehran, Iran) and cultured as described previously [10]. In brief, cells were maintained in α-modified Eagle's medium supplemented with 10% FBS, 1% penicillin/streptomycin, 1 mM sodium pyruvate and 100 mM non-essential amino acids at 37°C and a 5% CO_2_ environment.

### Preparation of nanoliposomes

To preparation of artemisinin and transferrin-loaded magnetic nanoliposomes with thermosensitive and non-thermosensitive properties, the SPC, DSPC, DPPC and CHOL with different molar ratios (Table 1) were dissolved in chloroform and thoroughly dried on a rotary evaporator (Brinkman, Toronto, Canada) under vacuum and N_2_ flow at 30°C. The dried lipids were dispersed by agitation in 6 mL of PBS-ethanol solution (v:v/5:1, pH = 7.4) containing artemisinin (12 mg), transferrin (12 mg) and magnetic iron oxide (12 mg) and then sonicated at 4°C in ultrasonic bath (Braun-sonic 2000, Burlingame, USA). Finally, artemisinin and transferrin-loaded magnetic nanoliposomes were obtained by extruding the respective suspension through a polycarbonate membrane with 100 nm-sized pores 12 times, and separating the excess artemisinin, transferrin and larger lipid aggregation by ultracentrifugation (100000 g, 30 min). Moreover, the non-trapped magnetic iron oxide was separated by a previously described method [11]. The control magnetic nanoliposomes were prepared similarly, but PBS (pH, 7.4) was used instead of the artemisinin and transferrin solutions. Before the preparation of nanoliposomes, the phase transition temperatures of used phospholipids were tested by a differential scanning calorimetric (DSC) method, as reported previously [12].

**Table 1:** Lipid composition of magnetic nanoliposomes

**Type of magnetic nanoliposomes**	**Lipids**	Molar ratio of lipids* (μmols/mL)
Non-thermosensetive	SPC:CHOL	30:6
Thermosensetive	DPPC:DSPC:CHOL	26:4:6

### Physiochemical characterization of nanoliposomes

#### Determination of encapsulation efficacy

The content of the artemisinin and transferrin in the nanoliposomes were determined by HPLC method following dissolution in 0.1% Triton X-100.

To determination of artemisinin, the 20 μL of nanoliposomal lysate was injected into the HPLC column. In the HPLC analysis, a C18 column (3.9 mm × 150 mm, 5 μm, Waters Co., Milford, USA) and diode array UV detector was used. The mobile phase was 2:1 (v:v) acetonitrile:water at a flow rate of 1 mL/min. The calibration curve was produced by diluting artemisinin stock solution in the mobile phase. To determination of transferrin, the 25 μL of nanoliposomal lysate was injected into a Polypropyl A HPLC column (PolyLC Inc., MD, USA). The column was eluted with a linear salt gradient from 2 M ammonium sulfate (pH 6.5) to 0.1 M potassium phosphate (pH 6.5) at a flow rate of 1 mL/min. The HPLC system was equipped with UV–vis detector (280 nm). The transferrin calibration curve was created by diluting its stock solution with mobile phase.

The content of magnetic iron oxide was determined using previously reported methods [13], with some modification. In brief, the magnetic iron oxide nanoparticles were separated using centrifugation after lyses of nanoliposomes. The precipitate was then dissolved in 0.1 N HCl solution under stirring and a 5 mL of the supernatant was dissolved in the 750 μL of sulfosalicylic acid dihydrate solution 10% (w/v). Subsequently, 750 μL of ammonia solution 25% (w/v) was added to the solution and was analysed by using spectra for the total iron complex at 425 nm. The absorbance of the diluted sample obtained from the magnetic iron oxide stock solution was used for standard curve preparation. Finally, the percentage of artemisinin, transferrin and magnetic iron oxide loading were then calculated as:

The amount of artemisinin or transferrin or magnetic iron oxide in nanoliposome × total volume tested × 100 / Total sample volume × Initial amount of artemisinin or transferrin or magnetic iron oxide.

### Particle size, zeta-potential and polydispersity index determination

The mean particle size, zeta-potential and polydispersity index of the magnetic nanoliposomes were determined using Malvern zetasizer (Malvern instrument, Worcestershire, UK) apparatus, as reported previously [14]. Each experiment was done in triplicate.

### Shape, surface morphology and magnetic properties

The size and structure of the thermosensitive and non-thermosensitive magnetic nanoliposomes that contained artemisinin and transferrin were analysed by cryo-transmission electron microscopy (cryo-TEM), as described previously [15]. Briefly, a grid was immersed in the nanoliposomal sample reservoirs at room temperature, blotted with blotting paper, drew the sample into the grid and vitrified in liquid ethane. Subsequently, the sample was transferred to liquid nitrogen for storage. Digital imaging was performed at 200 kV in a stage cooled by liquid nitrogen. Finally, size analysis was performed using ImageJ software (NIH, Bethesda, MD, USA). The magnetic properties of prepared nanoliposomes were measured using a vibrating sample magnetometer (Meghnatis Daghigh Kavir Co., Iran), as described previously [16].

### *In vitro* release study, thermosensitive behaviors and size stability

To determination of artemisinin, transferrin and magnetic iron oxide released from the nanoliposomes, a cellulose membrane (molecular weight cut-off of 8000 kDa, Membrane Filtration Products, USA) was mounted between the donor and receptor compartments. The donor medium consisted of 1 mL of each magnetic nanoliposomal formulation. The receptor medium consisted of 10 mL of citrate-phosphate buffer (0.1 M, pH 7.4). During the dialysis, the temperature was kept at 37°C. At pre-determined time intervals, between 2 to 12 hours, the amount of the released artemisinin, transferrin and magnetic iron oxide were then determined by the above described methods. To confirm whether the prepared nanoliposomes exhibit a thermal sensitivity, their stability in 42°C for 4 h was investigated. The size stability of artemisinin and transferrin-loaded magnetic nanoliposomes was assessed by measuring the particle sizes after 1 month storage at 4°C.

### *In vitro* efficacy

*In vitro* selective targeting of MCF-7 and MDA-MB-231 cell lines by prepared magnetic nanoliposomes was performed using previously reported methods [17], with some modification. Briefly, 100 μL of the MCF-7 and MDA-MB-231 cells (3 × 10^4^ cells/mL) was added into each well of a 96-well plate and allowed the cells to attach. In the absence or presence of a magnet, the proliferation of cells in the presence of different concentration of free and encapsulated artemisinin, transferrin and magnetic iron oxide for 12, 24, and 48 h was evaluated. Tamoxifen (7.43 μg/mL) was used as a positive control.

In order to evaluate the antiproliferative effects of free and encapsulated artemisinin, transferrin and magnetic iron oxide on MCF-7 and MDA-MB-231 cell lines in the presence of a magnetic field, a magnet (127.8 × 85.6 × 7 mm) was added to below the 96-well plate (with 24-well plate in between), and then the incubation was done.

At the end of the treatment, cells proliferation was analysed by 3-(4, 5-dimethylthiazol-2-yl)-2,5-diphenyl tetrazolium bromide (MTT) assay. In brief, 20 μL of MTT (5 mg/mL in PBS) was added to each well and samples were incubated for 4 h at 37°C. The MTT solution was removed, and 200 μL DMSO was added into each well to dissolve the precipitate. Then, optical density of the wells was measured at 570 nm.

### Data analysis

All data were expressed as means ± standard deviation (SD). The analysis of variance was performed to determine the significance level among the tested groups. The *P* values less than 0.05 were considered statistically significant.

## Results

**Figure 1:**
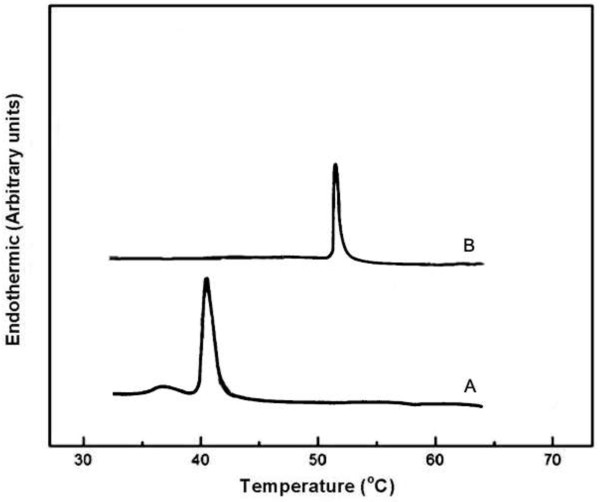
Phase transition temperature of DPPC and SPC.

The encapsulation efficacies of artemisinin, transferrin and magnetic iron oxide in the thermosensitive magnetic nanoliposomes were 83.06% ± 0.53, 80.12% ± 0.12 and 66.14% ± 0.42, respectively. Moreover, the encapsulation efficacies of artemisinin, transferrin and magnetic iron oxide in the non-thermosensitive nanoliposomes were 89.11% ± 0.23, 85.09% ± 0.31 and 78.10% ± 0.24, respectively.Our results showed that the phase transition temperature of DPPC (as the main phospholipid of thermosensitive nanoliposomes) was lower than SPC (Figure 1).

**Figure 2:**
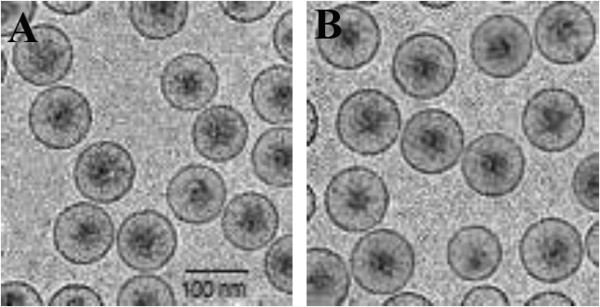
Cryo-transmission electron micrographs of the thermosensitive (A), and non-thermosensitive (B) magnetic nanoliposomes loaded with artemisinin and transferrin.

The particle size, zeta-potential and polydispersity index of the non-thermosensetive nanoliposomes were 99.12 ± 0.11, -1.25 ± 0.23 and 0.21 ± 0.05, respectively. Likewise, the particle size, zeta-potential and polydispersity index of the thermosensetive nanoliposomes were 95.06 ± 0.15, -1.40 ± 0.22 and 0.19 ± 0.09, respectively.Cryo-TEM analysis showed that the nanovesicles have a fine spherical shape and rough surface with a relatively monodispersed size distribution confirming the size distribution measurement studies (Figure 2).

**Figure 3:**
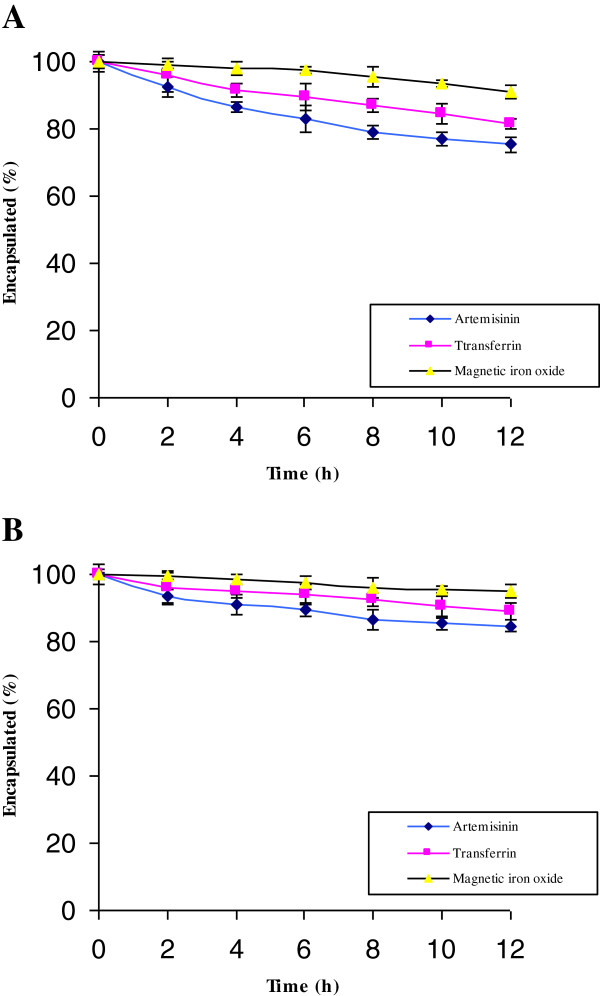
Percentages of artemisinin, transferrin and magnetic iron oxide recoveries for the thermosensitive (A) and non-thermosensitive (B) magnetic nanoliposomes at 37°C.

**Figure 4:**
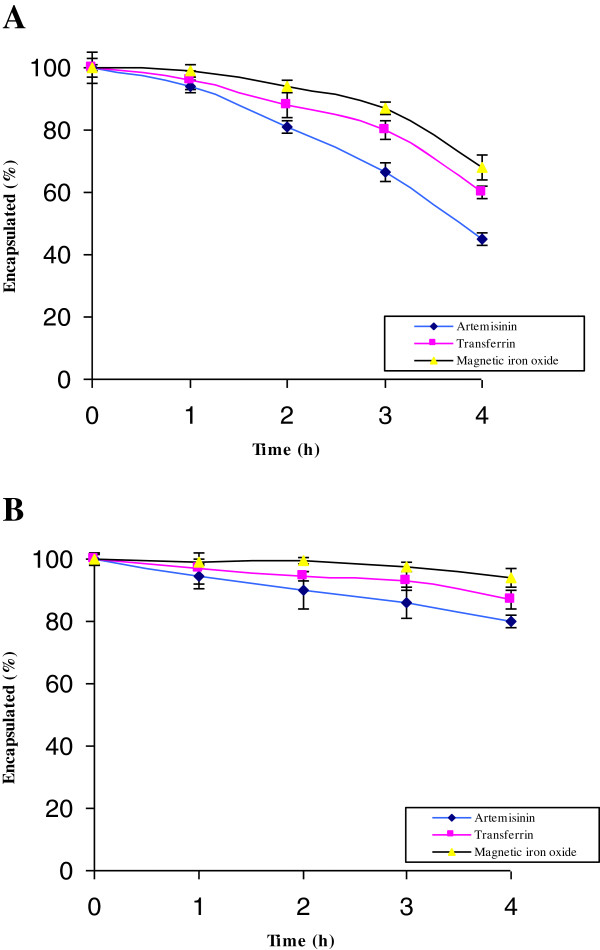
Percentages of artemisinin, transferrin and magnetic iron oxide recoveries for the thermosensitive (A) and non-thermosensitive (B) magnetic nanoliposomes at 42°C.

The saturation magnetizations for thermosensitive and non-thermosensitive nanoliposomes were 30.5 and 35.7 electromagnetic unit per gram (emu/g), respectively. In addition, there was no hysteresis in the magnetization with both remanence and coercivity, indicating that these magnetic nanoliposomes were superparamagnetic.The released amount of artemisinin, transferrin and magnetic iron oxide at 37°C from each nanoliposomal formulation were plotted as a function of time (Figure 3). The percentages of the artemisinin, transferrin and magnetic iron oxide recoveries for the thermosensitivity nanoliposomes after 12 h were 75.3% ± 2.1, 81.5% ± 2.3 and 91.1% ± 1.4, respectively (Figure 3A).The incubation was conducted at 42°C for 4 h to determine the extent of spontaneous and nanoliposomal-mediated artemisinin, transferrin and magnetic iron oxide released from the prepared nanoliposomes (Figure 4). The results showed an approximately 2-fold increase in artemisinin release from thermosensitivity nanoliposomes after 4 h at 42°C (Figure 4A).

**Figure 5:**
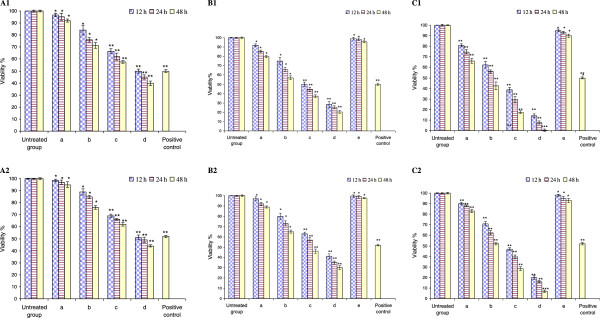
**Dose- and time-dependent inhibition of MCF-7 and MDA-MB-231 cellular growth by the combined free artemisinin, transferrin and magnetic iron oxide in the presence of an external magnetic force (A1 and A2), and by the thermosensitive artemisinin and transferrin-loaded magnetic nanoliposomes without (B1 and B2), and with (C1 and C2) an external magnetic force.** (**a**: Contained 12.50 μg/mL (44.27 μM) artemisinin, 12.80 μg/mL (0.16 μM) transferrin and 9.93 μg/mL (42.88 μM) magnetic iron oxide; **b**: 2 × **a**; **c**: 4 × **a**; **d**: 8 × **a**; **e**: control magnetic nanoliposones contained 9.93 μg/mL magnetic iron oxide). As a positive control, the tamoxifen (7.43 μg/mL, 20 μM) was used. Data were expressed as mean ± standard deviation from three independent experiments (*p < 0.05, **p < 0.01 and ***p < 0.001).

The physical stability of prepared nanoliposomes was evaluated by comparing different changes in mean diameters during their storage. Two formulations have minor size changes within 60 days storage at 4°C in PBS, so that the mean diameter of thermosensitive and non-thermosensitive nanoliposomes was 98.25 ± 0.14 and 106.20 ± 0.12 nm, respectively.In the presence or absence of an external magnetic field, the effect of the combination of artemisinin, transferrin and magnetic iron oxide in the free and encapsulated forms on MCF-7 and MDA-MB-231 cellular growth were examined by MTT assay. The results showed that the cell proliferation was inhibited in the MCF-7 and MDA-MB-231 cells in a dose- and time-dependent manner (Figure 5). Under identical conditions, the viability ratios of treated MCF-7 cells were lower than MDA-MB-231 cells. In all conditions, the artemisinin and transferrin-loaded magnetic nanoliposomes were more effective than those of free artemisinin, transferrin and magnetic iron oxide on MCF-7 and MDA-MB-231 cellular growth. As shown in Figure 5 C1 and C2, in the presence of magnetic field, the extent of inhibition increased significantly at 12 h with the lowest concentration of artemisinin, transferrin and magnetic iron oxide in the free and encapsulated forms which was continued to rise up, with 24 and 48 h durations at their maximum concentration.

## Discussion

The use of plant derived-loaded nanoliposomes for cancer therapy has been widely investigated [18,19]. The main problem associated with the application of such liposomal formulations is insufficient delivery to the target site [20]. As an interesting approach to drug delivery research, magnetic iron oxides were incorporated into nanoliposomes under the action of a magnetic field could overcome this limitation [21]. It is documented that the magnetic nanoliposomes that contained doxorubicin or adriamycin were tailored to target cancer cells, and the application of a magnetic field could increase drug concentration in the tumors [22,23].

Artemisinin is a sesquiterpene lactone and phytochemical found naturally in *Artemisia annua* L. [1]. Evidence for artemisinin’s benefit was strongest for anti-malaria, anti-oxidative, anti-inflammatory, and anti-cancer effects [2,5]. It is reported that the anti-cancer effect of artemisinin in the presence of iron sources such as transferrin was increased several fold [3].

In this study, we evaluated the potential of incorporating artemisinin and transferrin into magnetic nanoliposomes. We found that the encapsulation efficiencies of artemisinin and transferrin in the thermosensitivity nanoliposomes were suitable. According to the literature, this result was related to some condition, such as liposomal lipid content [24].

The liposomal particle size is highly dependent on the molar ratio of membrane lipids [25]. Our results showed that the increasing of the DPPC molar ratio could reduce the size of prepared nanoliposomes. Previous researches showed that the DPPC have excellent biocompatibility to form small nanoliposomes due to the ratio of head group size compared to hydrocarbon tail [26].

In this research, we found that the thermosensitive artemisinin and transferrin-loaded magnetic nanoliposomes produced a greater reduction in the proliferation of MCF-7 and MDA-MB-231 cells. This finding is in accordance with previous studies showing that the size of magnetic nanoliposomes is an important factor for their *in vitro* and *in vivo* distributions, pharmacodynamics and effectiveness [27].

The polydispersity index is an important indicator of the physical stability of nanoliposomes. The polydispersity index values between 0.1 and 0.25 indicate acceptable uniformity, while values >0.5 are indicative of poor uniformity [28].

Our results showed that the thermosensitive magnetic nanoliposomes have an acceptable polydispersity index, size homogeneity as well as the stability in pH 7.4 citrate-phosphate buffers within 12 h at 37°C. Therefore, in accordance with previous reports the best prepared thermosensitive magnetic nanoliposomes not only have an appropriate particle size and stability for cancer therapy but also have an appropriate size for use as a targeted therapeutic agent [29].

According to the literature, the change in the physicochemical properties of nanoparticles could alter their biokinetics parameters such as their toxicity and bioavailability [30].

Our study showed that the thermosensitive magnetic nanoliposomes have a more negative charge than another formulation. It has been reported that the interactions between the magnetic nanoliposomes and the some human cells such as capillary endothelium are zeta potential dependent [31]. Therefore, it revealed that the thermosensitive magnetic nanoliposomes would have a better potency for targeted therapy.

We found that the application of an external magnetic force could increase MCF-7 and MDA-MB-231 cellular death by artemisinin and transferrin. An approximately 12 h application of magnetic force elicits the maximum antiproliferation from thermosensitive magnetic nanoliposomes, as presently prepared. It was shown that the *in vitro* stability of magnetic nanoliposomes could also affect their performance [32], and our finding could explain this phenomenon. Magnetic artemisinin and transferrin-loaded nanoliposomes alone (without magnetic force) elicited higher antiproliferative activity on MCF-7 and MDA-MB-231 cells than free artemisinin, transferrin and magnetic iron oxide alone or in their combination. When nanoliposomes containing compounds were used *in vitro*, they can interact with the membranes of exposed cancer cells, and therefore decrease their viability and proliferation [33]. It is documented that the effect of the same nanoparticles on various cells is significantly different and could not be assumed for other cells [34]. Therefore, in accordance with "cell vision" effect, we noted that the prepared nanoliposomes have antiproliferative effects on the examined cells, and their efficacy for the other cell types may be varied.

## Conclusion

We have successfully prepared artemisinin and transferrin-loaded magnetic nanoliposomes in the thermosensitive and non-thermosensitive forms with acceptable uniformity, sustained release profile at 37°C and superparamagnetism. Current work demonstrated that artemisinin and transferrin-loaded magnetic nanoliposomes, in particular in the thermosensetive form have potent anti-growth effect on MCF-7 and MDA-MB-231 cells, and time-dependently inhibit cell growth in these cell lines. To the best of our knowledge, artemisinin and transferrin-loaded magnetic nanoliposomes treatment in combination with an external magnetic force resulted in an excellent decrease in proliferation of MCF-7 and MDA-MB-231 cells. Therefore, these novel formulations could be a promising approach for artemisinin and transferrin targeted cancer therapy.

## Competing interests

The authors declare that they have no competing interests.

## Authors’ contributions

All authors have read and approved the final manuscript.
